# Use of a Rapid Qualitative Method to Inform the Development of a Text Messaging Intervention for People With Serious Mental Illness Who Smoke: Formative Research Study

**DOI:** 10.2196/40907

**Published:** 2022-11-07

**Authors:** Catherine S Nagawa, Ian A Lane, Colleen E McKay, Ariana Kamberi, Lisa L Shenette, Megan M Kelly, Maryann Davis, Rajani S Sadasivam

**Affiliations:** 1 Department of Population and Quantitative Health Sciences UMass Chan Medical School Worcester, MA United States; 2 Tan Chingfen Graduate School of Nursing UMass Chan Medical School Worcester, MA United States; 3 Department of Psychiatry UMass Chan Medical School Worcester, MA United States; 4 VISN 1 New England Mental Illness Research, Education, and Clinical Center VA Bedford Healthcare System Bedford, MA United States

**Keywords:** serious mental illness, mental disorder, psychiatric disorder, tobacco use, smoking cessation, text messaging, intervention, smoking, mental health, virtual, COVID-19, pandemic, symptom

## Abstract

**Background:**

People with serious mental illness are disproportionately affected by smoking and face barriers to accessing smoking cessation treatments in mental health treatment settings. Text-based interventions are cost-effective and represent a widely accessible approach to providing smoking cessation support.

**Objective:**

We aimed to identify key factors for adapting text-based cessation interventions for people with serious mental illness who smoke.

**Methods:**

We recruited 24 adults from mental health programs who had a serious mental illness and currently smoked cigarettes or had quit smoking within the past 5 years. We then conducted virtual qualitative interviews between November 2020 and August 2021. Data were analyzed using the rapid thematic analytic approach.

**Results:**

We identified the following 3 major themes: (1) interplay between smoking and having a serious mental illness, (2) social contextual factors of smoking in adults with serious mental illness, and (3) smoking and quitting behaviors similar to the general population. Participants reported barriers and facilitators to quitting across the 3 themes. Within the “interplay between smoking and having a serious mental illness” theme, barriers included smoking to manage stress and mental health symptoms, and facilitators to quitting included the awareness of the harm of smoking on mental health and patient-provider discussions on smoking and mental health. In the “social contextual factors of smoking in adults with serious mental illness” theme, barriers included high social acceptability of smoking among peers. Positive support and the combined social stigma of smoking and having a mental health condition outside of peer groups motivated individuals to quit. Some participants indicated that low exposure to other smokers during the COVID-19 pandemic helped them to engage in cessation efforts. In the “smoking and quitting behaviors similar to the general population” theme, barriers included smoking after eating, having coffee, drinking alcohol, and experiencing negative social support, and facilitators included health concerns, improvement in the general quality of life, and use of evidence-based tobacco treatments when available.

**Conclusions:**

People with serious mental illness often smoke to cope with intense emotional states, manage mental health symptoms, or maintain social bonds. Text message content emphasizing equally effective and less harmful ways for stress reduction and mental health symptom management may improve quit rates in individuals with serious mental illness.

## Introduction

People with serious mental illness report a higher smoking prevalence (41%) than people without serious mental illness (14%) [1,2]. Unfortunately, most people with serious mental illness who smoke have little or no access to pharmacotherapeutic cessation aids and behavioral approaches, which are the recommended treatments for smoking cessation [3]. While mental health treatment settings offer a possibility for engaging people with serious mental illness in smoking cessation, only a minority of them are currently receiving treatment [4]. In 2019, 46% of the 52.9 million US adults with any mental illness had received mental health services in the past year [4]. Further, many mental health settings do not routinely offer tobacco treatment [5]. Alternative approaches to engage and treat people with serious mental illness for smoking cessation are needed.

Text-based interventions are cost-effective and eliminate many barriers to accessing traditional treatments [6,7]. Ninety percent of individuals with a mental health diagnosis own more than one mobile device, including a mobile phone, and many report high use of multiple devices [8]. In the general population, text-based cessation interventions significantly improve quit rates and use of telephone counseling (ie, Quitline) services [9]. Studies have shown the feasibility of text-based cessation interventions in people with serious mental illness who smoke [10-12]. However, text-based cessation interventions need to address barriers to quitting that are specific to people with serious mental illness.

In the literature, many factors have been noted for increased tobacco use rates among people with serious mental illness. For instance, people with mental health conditions report using cigarettes to alleviate emotional problems, such as depression and anxiety, stabilize their mood, and relieve stress [13-15]. Many of these factors are shared with the general population of people who smoke. However, studies have not fully explored how these factors and mental health conditions intersect to increase tobacco use rates and quitting challenges in people with mental illness. Adapting text-based cessation interventions to address this intersection directly may increase their relevance and effectiveness, and improve engagement among people with serious mental illness who smoke [16].

The effectiveness of the text-based messaging intervention being adapted has been demonstrated in a large randomized trial conducted among individuals without serious mental illness [17,18]. Our intervention included use of expert and peer-written messages [19]. We developed the expert-written messages through an iterative group review process guided by theoretical frameworks and existing smoking cessation guidelines [3]. Peer-written messages were written by current and former smokers responding to an online survey. The content of expert messages was more “biomedical” in nature (avoidance, behavioral strategies, and health), while the content of peer messages focused on “social” and “real-life” aspects of smoking (expectations, money, quality of life, attitudes, and friends) [19]. The intervention group received motivational text messages weekly for 6 months, and compared with the control group, receiving text messages increased the odds of 7-day point prevalence cessation at 6 months (odds ratio 1.69, 95% CI 1.03-2.8) [17,18]. However, these messages do not address barriers associated with serious mental illness.

In this study, we explored tobacco use behaviors and barriers along with facilitators of quitting among 24 people with serious mental illness to identify key factors for adapting a text messaging intervention that addresses their cessation needs. We also detailed a 4-step approach to identify themes from the qualitative interviews that could be a structured process for future text-based intervention developers.

## Methods

### Study Overview

We conducted an in-depth qualitative exploration of smoking behaviors, barriers, and facilitators among adults with serious mental illness who currently smoke or quit within the past 5 years.

We used a 4-step approach to identify key themes that will inform the development of text messages targeted at people with mental health conditions. Briefly, the 4-step approach included identifying domains that aligned with key interview questions, summarizing transcript data under each domain, consolidating the data into a matrix, and interactively identifying themes under each domain. We provide details in the data analysis section.

### Study Setting and Participant Recruitment

Participants were recruited nationally from 3 mental health agencies, most of which are publicly funded (Clubhouses accredited by the International Center for Clubhouse Development [20], Thresholds Agency, and Massachusetts Department of Mental Health agencies such as Open Sky). These mental health programs provide services to individuals with various mental health conditions. Services include the opportunity for friendship, employment, housing, education, and access to medical and psychiatric services. Clubhouses are accessed by about 100,000 people with mental health conditions [21]. Thresholds is a large community mental health center in Chicago that serves 12,500 adults and youth each year. The Massachusetts Department of Mental Health programs and Clubhouses affiliated with Clubhouse International provide various clinical and nonclinical services to adults with serious mental illness.

We recruited 24 adults with serious mental illness through virtual information sessions administered by a research coordinator. Individuals were eligible to participate if they (1) were 18 years or older, (2) were currently smoking cigarettes or had quit within the past 5 years, (3) were willing and able to provide consent, and (4) were currently receiving services from mental health agencies (ie, Clubhouses, Thresholds Agency, and Massachusetts Department of Mental Health agencies such as Open Sky). We excluded pregnant individuals or those attending correctional programs (ie, prisoners).

### Ethical Considerations

Potential participants provided contact information to the research coordinator, who set up one-on-one virtual meetings to provide more information about the study. Those interested in participating provided informed electronic consent (e-consent) before enrollment into the study. Electronic signatures were captured using the Research Electronic Data Capture e-consent framework. Participants received a US $15 Amazon electronic gift card for the study. This project was approved by the ethics review boards at the Department of Mental Health in Massachusetts and the University of Massachusetts Chan Medical School (reference number: H00019687_3).

### Interview Guide Development

The interview guide was developed by researchers with expertise in mental health and tobacco use based on prior literature [22-24]. The interview guide was structured using domains that aligned with the interview questions and the overall study objective of developing text messages for people with serious mental illness. We chose the approach outlined in *Rapid qualitative inquiry: A field guide to team-based assessment* by James Beebe [25] to generate the domains explored in our study. Informed by the literature, the research team identified the key factors that would be the most meaningful in informing the implementation aspects of a smoking cessation study. Such factors included the context in which individuals with serious mental illness smoked, barriers and facilitators to quitting, and how one’s mental health diagnosis may influence their smoking habits. These factors guided the formulation of the key research questions/interview guide, which shaped our study domains.

The final interview questions broadly focused on motivations, beliefs, and barriers and facilitators to smoking or quitting. We also asked about smoking triggers or cues; symptoms of tobacco dependence; previous experiences reducing or stopping smoking, including use of and barriers to evidence-based cessation; and the role of interpersonal relationships in smoking cessation. During the interviews, we prompted participants to reflect on how their diagnosis has influenced their smoking behaviors and quitting experiences. The specified 9 domains that aligned with these questions included the context in which participants smoked, smoking triggers, reasons for smoking, barriers to active quitting, facilitators of active quitting, mental health–specific quitting strategies, mental health–specific facilitators of quitting maintenance, and the influence of the COVID-19 pandemic on smoking.

We refined the interview guide based on feedback from a stakeholder advisory panel of 9 adults with serious mental illness who also currently smoked or had quit within the past 5 years. Panel members were recruited from the same mental health agencies as study participants to ensure the representativeness of the study population.

### Data Collection

Two trained researchers conducted the qualitative interviews using a semistructured interview guide via Zoom video conference software (Zoom Video Communications Inc). Interviews were conducted between November 2020 and August 2021, and lasted for 30 to 40 minutes. All interviews were audio recorded, deidentified, and professionally transcribed. In addition to the qualitative interview, participants completed a baseline questionnaire that captured demographic information (eg, age, sex, race/ethnicity, and educational level) and smoking behavior or history of those who recently quit.

### Data Analysis

We used the rapid thematic qualitative analytic approach to analyze qualitative data. This rapid approach is a team-based qualitative inquiry that uses triangulation and iterative data analysis to quickly develop a preliminary understanding of a situation from the insider’s perspective [25-27]. This approach to data analysis provided insights into the context in which participants smoked and their attitudes and beliefs toward quitting smoking.

The 4-step process is as follows:

1. We used the domains developed in the interview development step to create a transcript summary template in Microsoft Word (Microsoft Corp). The summary template was structured so that each section on the template corresponded to a domain. The summary template guided the analysis team in mapping each participant’s responses to the corresponding domain.
2. We performed a “test drive” to assess the domains’ usability, relevance, and consistency. In this step, 2 coders (CSN and IAL) used the template to summarize the same transcript to evaluate whether the specified domains were identifiable in the data and to check for consistency across coders in capturing the domains. We modified the template based on feedback from the rest of the research team (RSS, MD, AK, and CEM) before testing with a second transcript. Once consistency was established, we divided the transcripts equally across the 2 coders and summarized the transcripts using the modified template.
3. We transferred transcript summaries into a matrix. We placed information from the 24 transcript summaries into a Microsoft Excel matrix. The transcript summaries were put into a matrix to analyze each domain’s depth and breadth of data [25]. Each column in the Excel document represented a prespecified domain, and the rows represented study participants. The cells in the Excel document contained summarized responses from each study participant that corresponded to a specific domain.
4. We identified study themes and subthemes using the matrix. Themes were identified within each domain. The research team collaboratively and iteratively reviewed, discussed, and sorted the data to refine the initial themes and subthemes, and highlight the most salient quotes. The research team members provided multiple perspectives; a triangulation (investigator triangulation [28]) approach was designed to increase the findings’ reliability. In addition to investigator triangulation, we used the peer-review approach [29], in which a researcher (MMK) who was not involved in data collection reviewed the evidence that supported the interpretation of the data and conclusions. The final themes highlight the views and experiences with using family or peer support for smoking cessation and considerations for support of cessation interventions. A brief descriptor (subtheme) of what participants reported is provided within each theme. Each of these themes will inform the text message content.

## Results

### Participant Characteristics

Participant characteristics are presented in Table 1. Most participants were between 36 and 54 years old (11/24, 46%) and male (15/24, 63%). About half self-identified as non-Hispanic White (13/24, 54%) and had a high school education (13/24, 54%). More than half (10/17, 59%) of the participants who were currently smoking had made a quit attempt in the past year. A high proportion (20/24, 83%) of study participants owned a cell phone or smartphone, and a majority (19/20, 95%) of those who owned a cell phone or smartphone often used it to send and receive text messages (Table 1).

**Table 1:** Sociodemographic characteristics of the study participants.

Participant characteristic				Value (N=24), n (%)
**Age (years)**				
	18-34			9 (37%)
	36-54			11 (46%)
	55-65			4 (17%)
**Gender**				
	Male			15 (63%)
	Female			8 (33%)
	Nonbinary			1 (4%)
**Race and ethnicity**				
	Non-Hispanic White			13 (54%)
	Non-Hispanic Black			5 (21%)
	Other			6 (25%)
**Education level**				
	Some high school/high school graduate			13 (54%)
**How hard is it for you (and your family) to pay for medical care?**				
	Very hard			3 (12%)
	Somewhat hard			4 (17%)
	Not very hard			16 (67%)
	Don’t know			1 (4%)
**Do you take medication for your mental health?**				
	Yes			23 (96%)
**Do you currently smoke?**				
	**Yes**			17 (71%)
		Past 12 months quit attempts among those who currently smoke (N=17)	10 (59%)	
**Past 30-day electronic cigarette use**				
	Every day or some days			6 (25%)
	Not at all			18 (75%)
**Is smoking allowed where you live?**				
	Yes			20 (83%)
**Besides yourself, does anyone who lives with you smoke cigarettes now?^a^**				
	No			16 (70%)
	Yes			7 (30%)
**Do you own a cell phone or smartphone?**				
	Yes			20 (83%)
**Do you ever use your cell phone to send or receive text messages? (N=20)**				
	Yes			19 (95%)

^a^Information is missing for 1 participant.

### Factors for Adapting Text-Based Interventions for People With Serious Mental Illness Who Smoke

We found 3 major themes that provided insights into the experiences of quitting smoking in people with serious mental illness. The themes included interplay between smoking and having a serious mental illness, social context of smoking, and similarities in smoking and quitting behaviors between the participants and the general population (Table 2). There were barriers and facilitators within each theme, as presented below.

**Table 2:** Factors for adapting text-based interventions for people with serious mental illness who smoke.

Variable	Theme 1: Interplay between smoking and having a serious mental illness	Theme 2: Social contextual factors of smoking in adults with serious mental illness	Theme 3: Smoking and quitting behaviors similar to the general population
Smoking patterns	Smoking occurs when anxiety, stress, depression, or nervousness is heightened	Individuals tend to smoke with friends and at social events (or parties)	Smoking is habitual; usually done after eating or having coffee, or when drinking alcohol
Reasons for smoking	Smoking is perceived to improve mood and reduce anxiety	Smoking is perceived to help with social interactions or be part of social activities	Stress management
Motivations to quit smoking	Negative impact on mental health	The social stigma associated with being a smoker and having a mental health condition	Motivated to quit for esthetic reasons Negative impact on physical health Financial cost of smoking
Challenges of quitting	Ways to manage mental health when quitting	Socially acceptable or rewarding to smoke	Negative or counter-productive support Boredom and cigarette sharing
Facilitators of quitting	Mental health care providers	Positive social support	Use of evidence-based cessation approaches
Impact of the pandemic on quitting	Smoking continued in part as a coping response to the stress/anxiety of the pandemic	Lower levels of exposure to other individuals who smoke facilitated cessation	The pandemic did not add to the motivation to quit for those who perceived their risk of COVID-19 to be low

### Theme 1: Interplay Between Smoking and Having a Serious Mental Illness

#### Barriers

Individuals often smoked when mental health symptoms (such as feelings of anxiety, stress, or nervousness) were heightened, and the perception was that smoking helped with these symptoms.

Participants said:

Because I know that when I'm having a bipolar episode, and I started having voice issues and more paranoia and stuff like that, something about having a cigarette just calms it down.

Female, 45-54 years old, currently smokes

Anytime, like anytime someone raised their voice with me. Anytime I thought I might be in trouble. Anytime I had to get something done, but time was short. Anytime I had to make a phone call. Phones always used to cause me anxiety. Anytime I had to… Gosh, the exhaustion of having to make dinner. Oh, my gosh. I better just go smoke, you know?

Female, 35-44 years old, recently quit

One participant who found smoking helpful in managing mental health symptoms said:

I mean any time that I get depressed, I'm able to like to go out and have a cigarette. And it just seems like as I'm smoking, like all my worries just go away because it's letting me calm down rather than getting worse.

Female, 21-23 years old, currently smokes

Smoking continued in part as a coping response to the stress and anxiety induced by the COVID-19 pandemic.

When COVID hit, it just made it worse. I wanted to drink. I'm trying to drown my sorrows away. Like I'm depressed. I'm sad for the people that's like literally dying. They didn't… You know what I'm saying? They didn't think it was going to be their time yet. It was just so much death. Everybody was getting sick. It made it intensify. Like, oh, my God. I gotta smoke. I gotta have a drink. I gotta smoke. You know what I'm saying? Like I gotta have a cigarette. I'm stressed out. The world's stressed out. The world's goin' crazy. The world's ending! Like it has affected me. I'm not gonna lie.

Female, 30-34 years old, currently smokes

#### Facilitators

Participants were motivated to stop smoking due to the awareness of the negative impact of smoking on their mental health. Specifically, 2 participants said:

So, it wrecks my health, because my mental health and my physical health are very, very intertwined. So, I turn into an anxious monster because the moment I extinguish a cigarette, my body goes into withdrawal, and I just want another cigarette. And then I'm looking and hunting for that, which for me produces a lot of anxiety.

Female, 35-44 years old, recently quit

I think sometimes it makes [mental health diagnosis] worse. Yeah, the smoking makes like my anxiety worse sometimes, also. cause it gets my heart goin'. And then if I can't like to control my breathing, but then sometimes it's also the opposite of where it just takes me away from everything.

Male, 24-26 years old, currently smokes

Although managing mental health symptoms during cessation was a significant barrier to successful quitting, participants found that speaking with their mental health care provider helped them navigate the quitting process better.

I even tried Chantix, which my psychiatrist was very wary about prescribing me because of my history with suicidality. But I was, like, in a really good place now. And you know, I'm always honest about my feelings. So, can we give it a try? And then if it does make my symptoms worse, we'll just take me off of it right away. And I didn't even last seven days on it before I started feeling suicidal. So, I couldn't even take Chantix.

Female, 45-54 years old, currently smokes

I know at one point we tried Wellbutrin for both mental health and smoking, and that was one I don't think had any effect on me. I am currently waiting to see if quitting smoking has an effect on my sleep, which would then have an effect on my psychiatric sleep medication. But we haven't quite got there yet because I have to get off some of my Chantix to be able to see that. So that's the big one that we're waiting to see if my sleep gets better now that I'm not smoking because that smoking causes some bad sleep apnea.

Female, 35-44 years old, recently quit

### Theme 2: Social Contextual Factors of Smoking in Adults With Serious Mental Illness

The social context played a vital role in the smoking and quitting behaviors of people with serious mental illness.

#### Barriers

Smoking seemed socially acceptable among peers and was viewed as a social bonding activity.

Individuals often smoked during social gatherings. Participants said:

Yeah, when you go out there, there's like two or three picnic tables in the winter or the summer, and there's like maybe 10 smokers or 15 smokers. So, you join in with the crowd and you don't feel left out. I feel more blended in with them because they smoke.

Male, 55-65 years old, recently quit

If I'm trying to have a conversation, I feel a little uneasy without a cigarette, but I'm getting much better about that. But it used to be, if I am, you know, out on a patio with friends, it doesn't feel right unless I'm smoking. I'm getting better with that. But yeah, social situations. A cigarette always makes you feel like you've got your best friend there.

Female, 35-44 years old, currently smokes

Smoking seemed socially rewarding. Some individuals used smoking to feel socially included or make friends. Participants said:

Obviously, smoking cigarettes can start friendships or bring people together in the first place because you might be out somewhere. Say you're going to a bar or something and you go outside, and everybody's had a few, and then you smoke a cigarette. You might get to know somebody you don't.

Male, 35-44 years old, recently quit

I know there were certain times I met people because we'd go… I'd go to smoke, and they were there, and it was like oh, I didn't know you smoked. Oh, hi. Hi. And then suddenly, you're friends. But there are better ways to meet people. So, see everything I say is really over time I've learned that there really is no good reason to smoke. Not that I shame anyone for doing it.

Female, 35-44 years old, recently quit

#### Facilitators

Participants were motivated to stop smoking due to the existing social stigma of smoking outside their peer networks and viewed positive support as a facilitator to quitting smoking. For instance, 1 smoker indicated that the social stigma of smoking, when added to the stigma of having a mental illness, was a motivator to stop smoking.

There's a stigma with smokers, you know? And there's a stigma with mental illness. I don't like stigma. I don't like labels. I don't like boxes, you know? I'd rather be a nonsmoker in peoples' eyes if I had to choose.

Female, 45-54 years old, recently quit

Social support also facilitated successful quitting. One participant said:

I think as I just got older, there were just more people in my life, who would say, you know, you should really stop smoking. You know, doctors, family members, friends who didn't smoke. It's just like there were more of those types of people in my life. And so, even though I didn't necessarily listen to them right then and there. In a way, I really did listen to them. I just kinda put it into the back of my mind. And I just eventually was like, you know, you don't really need this.

Male, 45-54 years old, recently quit

While some participants responded that social isolation increased their smoking behaviors, those who had low exposure to other smokers due to social distancing were able to engage in cessation efforts.

I wasn't necessarily even really enjoying it as much. Maybe because I was alone, and there wasn't that like, because of the pandemic, there wasn't that like social component to it, that made it even easier, I guess, to maybe quit I guess.

Male, 45-54 years old, recently quit

### Theme 3: Smoking and Quitting Behaviors Similar to the General Population

There were similarities in smoking and quitting behaviors between people with serious mental illness and the general population.

#### Barriers (Smoking Behavior)

Similar to the general population, participants tended to smoke out of habit, such as after eating or having coffee, or when drinking alcohol.

It's like clockwork. You have to smoke right after you eat. I don't even know why. I don't even think I did it because of any real craving or anything. It's like something you just become used to doing.

Male, 35-44 years old, recently quit

Like something about coffee and cigarettes, like I don't know. For me, they go together well. Like you know, you're trying to boost your energy up.

Female, 30-34 years old, currently smokes

Participants also smoked due to boredom or having easy access to cigarettes through friends.

Time, having time. I've had to take a lot of time to find other routines and other things to have in my hands. Especially as someone who is unemployed and disabled, it's like I don't need to be sitting around smoking. We have to do other things.

Female, 35-44 years old, recently quit

Because everybody here is smoking, you know, cigarettes. Like there's a guy here, and he buys, you know, a pound of tobacco at a time. And he'll roll a bunch, you know, with the tubes. And so he just hands them out. It's like very communal.

Male, 24-26 years old, currently smokes

Similar to the general population, negative social support made it challenging to quit. One smoker said:

And then I didn't really have anybody supporting me in it. I have family telling me that they didn't think I was gonna succeed. I didn't have anybody like supporting me, and saying, yeah, you can do it. I didn't have anyone like that. Just people saying, when are you gonna start smoking again?

Female, 45-54 years old, currently smokes

#### Facilitators (Quitting Experiences)

However, individuals had concerns about the negative impact of smoking on their physical health and general quality of life, which motivated them to quit smoking.

You know, again, I guess I was thinking down the line like I can't keep doing this until I'm 40 or 50. And so it was more, again, like thinking of my health down the road, as opposed to right now. And so, it was basically thinking long-term, I can't keep this up. I don't want to, you know, have any cancer or risk of heart attack, stroke, or heart disease.

Male, 30-34 years old, recently quit

In your clothing, in your hair. Everywhere around you, you know. Everything smells like… well, especially you come back home from fresh air from outside, it's just… or when you came out of shower and you walk in the room, it's just disgusting.

Male, 55-65 years old, currently smokes

Participants used evidence-based tobacco treatments and behavioral approaches to quit smoking.

Yeah, I've tried Chantix a couple of times. I used to not really believe in the patch or the gum up until, you know, recently.

Male, 24-26 years old, currently smokes

I have the Nicotrol inhaler. It's a prescription quit-smoking thing that's not available over the counter yet for some reason. I've tried it with the gum. I've tried with the patch. I think the Nicotrol inhaler is more effective because you're still puffing on something. So the Nicotrol inhaler's more helpful because you're puffing on something. So that was the only thing I was ever able to quit with at all.

Female, 45-54 years old, currently smokes

## Discussion

### Main Study Findings

Using the 4-step analytical approach, we identified the following 3 key aspects that can inform the development of text-based cessation interventions for people with serious mental illness who smoke: (1) interplay between smoking and having a serious mental illness, (2) social contextual factors of smoking, and (3) smoking and quitting behaviors similar to the general population. Across the 3 themes, participants reported barriers, motivators, and facilitators to quitting smoking. The variations in the content provided by the participants can facilitate the development of messages that can be implemented in text-based interventions targeted at people with serious mental illness who smoke.

Participants strongly believed that smoking helped manage mental health symptoms and was often used to cope with stress. Stress is often cited as a reason for smoking in individuals with and without serious mental illness [30,31]. However, because stress has a strong positive correlation with mental illness [32], people with serious mental illness may not progress through the stages of change at a rate comparable to that in those without serious mental illness. In addition, the term “stress” could be used as a proxy for mental health symptoms in this patient group. In such a case, smoking could be used as self-medication to alleviate feelings of intense emotions or to feel an immediate sense of relaxation even though individuals are aware of the harmful effects of smoking on their mental and physical health.

Individuals found that speaking with their mental health care provider when quitting helped them navigate the quitting process better. In discussions with their providers, they expressed the impact of tobacco medication (ie, Chantix) on their mental health symptoms. This finding implies that patient-provider discussions regarding smoking and mental health may facilitate successful quitting. Therefore, engaging health care providers when making a quit attempt is essential in this population. Patient-provider discussions regarding smoking also provide a critical opportunity for health care providers to monitor for smoking/quitting-drug interactions [33] as patients may not be aware of these interactions.

Adapting text-based cessation interventions to address barriers to smoking among people with serious mental illness may increase the intervention’s relevance, engagement, and effectiveness. Past text-based cessation interventions have not included content specific to mental health conditions [34]. A systematic review of text-based cessation interventions found that message content focused on increasing self-efficacy and encouraging smokers to quit or maintain their quit status by providing quit tips [34]. This content is very similar to the one we used in our own past studies [35,36]. Currently, text messages used in cessation interventions lack content that highlights mental health symptoms and stress management, and content that emphasizes the need to engage mental health care providers when quitting. Therefore, text messages that offer tips on evidence-based strategies for managing stress, regulating emotions, managing negative affect, and dealing with unhelpful thoughts/anxiety can be a meaningful inclusion in text-based interventions. Messages that serve as reminders to engage mental health care providers when quitting may increase the relevance of text-based cessation interventions in this patient population.

We found a strong tendency to smoke in people with mental health conditions and in the mental health environments in which they were embedded. From our observations, smoking seemed ingrained in all aspects of their social lives. For instance, smoking was perceived as a social bonding activity so much that individuals often perceived smoking as a way to be socially included or form friendships. Given the high level of social acceptance observed in this patient population [24,37], these barriers to quitting smoking extend beyond those experienced by people in the general population who smoke. Recognizing the broader factors that drive smoking rates within the living and social environments of people with serious mental illness can complement existing efforts aimed at helping them quit successfully. A few past text messaging interventions have included messages with content relevant to the social context [38]. The messages are about seeking social support or how to deal with a partner who smokes. Additional content could include messages that denormalize smoking [39], provide alternatives to smoking as a group bonding activity that could counteract the perceived social rewards of smoking, and provide information on how to identify cessation role models.

In addition to the distinct challenges identified by people with serious mental illness, they also face challenges commonly observed among people in the general population who smoke. Participants often smoked out of habit, such as after a meal or while drinking alcohol, due to boredom and having access to cigarettes. They reported similar reasons for quitting, including health benefits, the financial cost of cigarettes, and the general improvement in quality of life. Participants also used evidence-based strategies to quit smoking, including nicotine replacement therapy and tobacco treatment medications. These findings are similar to past study findings [40], which indicate that people with serious mental illness are motivated to quit and can engage in cessation using evidence-based resources, yet they often cannot quit. It is important to modify existing text-based interventions to provide information on mental health service access and to include content that increases antismoking attitudes.

Other researchers have discussed the need for a structured process to support the development of text messages [41]. The approach we used in this paper provided an efficient way to identify key factors that can inform text message development from formative interviews. We chose the rapid qualitative approach over other qualitative data analysis approaches because it provided a systematic way to describe the lived experiences of people with serious mental illness who smoke within each domain (eg, the context in which participants smoked, smoking triggers, reasons for smoking, barriers to active quitting, and facilitators of active quitting).

### Limitations

This study had limitations. Our study recruited participants from mental health agencies, who had access to mental health services. In many ways, individuals with access to mental health services could differ from those without access to mental health services regarding smoking behaviors, attitudes toward quitting, and cessation experiences. Targeting people with serious mental illness without access to health services could provide additional insights into these patients’ challenges. Additionally, we relied on self-reported information when assessing smoking status.

### Conclusion

People with serious mental illness may experience life stressors more intensely than those without serious mental illness. As a result, they often use smoking to cope with intense emotional states, manage mental health symptoms, or maintain social bonds. Based on our study findings, text message content should be modified to (1) provide information on the effect of smoking on mental health and alternative approaches to managing mental health symptoms, (2) incorporate smoking denormalization strategies that increase antismoking attitudes, and (3) provide healthier alternatives to smoking for social bonding activities. Adapted text-based interventions can ensure that people with serious mental health illness receive the appropriate support to stop smoking.

## Data Availability

This article’s data can be shared on reasonable request to the project principal investigators (MD and RSS).
